# Improvement of Growth, Yield, Seed Production and Phytochemical Properties of *Satureja khuzistanica* Jamzad by Foliar Application of Boron and Zinc

**DOI:** 10.3390/plants10112469

**Published:** 2021-11-16

**Authors:** Hasan Mumivand, Parisa Khanizadeh, Mohammad Reza Morshedloo, Edyta Sierka, Krystyna Żuk-Gołaszewska, Tomasz Horaczek, Hazem M. Kalaji

**Affiliations:** 1 Department of Horticultural Science, Faculty of Agriculture, Lorestan University, Khorramabad 68151-44316, Iran; parimail1989@gmail.com; 2 Department of Horticultural Science, Faculty of Agriculture, University of Maragheh, Maragheh 83111-55181, Iran; morshedlooreza@gmail.com; 3 Faculty of Natural Sciences, Institute of Biology, Biotechnology and Environmental Protection, University of Silesia in Katowice, 28 Jagiellonska, 40-032 Katowice, Poland; edyta.sierka@us.edu.pl; 4 Department of Agrotechnology and Agribusiness, Faculty of Agriculture and Forestry, University of Warmia and Mazury in Olsztyn, Oczapowskiego 8, 10-719 Olsztyn, Poland; kzg@uwm.edu.pl; 5 Institute of Technology and Life Sciences—National Research Institute, Falenty, Al. Hrabska 3, 05-090 Raszyn, Poland; t.horaczek@itp.edu.pl (T.H.); hazem@kalaji.pl (H.M.K.); 6 Department of Plant Physiology, Institute of Biology, Warsaw University of Life Sciences SGGW, 02-776 Warsaw, Poland

**Keywords:** essential oil, carvacrol, antioxidant property, seed germination

## Abstract

*Satureja khuzistanica* Jamzad is a valuable and endemic medicinal plant. Boron and zinc are essential elements for the vegetative and reproductive growth of plants and have significant effects on yield, essential oil composition and the seed production of plants. To investigate the effects of the foliar application of zinc and boron on the growth, yield, seed production and phytochemical properties of *S. khuzistanica*, a study was conducted in a factorial experiment with three replicates in two consecutive years based on a randomized complete block design. The foliar application of boron (B) at three concentrations (control or distilled water, 0.4% and 0.8% as H_3_BO_3_) and zinc (Zn) at three concentrations (control or distilled water, 0.3% and 0.6% as ZnSO_4_) was carried out. Our results showed that the foliar application of B resulted in a significant increase in the fresh and dry weights of plants, the dry weight of stems, drug yield, seed yield, seed germination and 1000-seed weight. At the same time, the application of B resulted in a significant decrease in seed emptiness. The fresh and dry weights of plants, drug yield, seed yield, 1000-seed weight and seed germination were also significantly improved by Zn foliar spraying compared to the control. Application of 0.8% B resulted in a significant decrease in seed emptiness by 14.16% and 22.37%, as compared to the control. The foliar spraying of B and Zn improved the total phenolic content, the essential oil content and the yield and antioxidant activity of *S. khuzistanica*. Moreover, B application generally concentrated more carvacrol in the essential oil (in the first experimental year). In contrast, no significant differences were observed between Zn treatments in carvacrol content and total flavonoids. The use of several microelements, such as B and Zn, could improve both the quantity and quality of *S. khuzistanica*. Additionally, improvement of seed set and seed quality by the foliar spraying of Zn and B may be useful for growing plants in arid and semi-arid areas.

## 1. Introduction

*Satureja khuzistanica* Jamzad, which belongs to the *Lamiaceae* family, is one of the most important medicinal plants endemic to Iran. Here, the plant grows wild in dry areas, sunny areas and the calcareous rocky soils of the southwestern region (Lorestan, Ilam and Khuzestan provinces) [1]. In Iranian folk medicine, *S. khuzistanica* is a popular herb for local people, used to treat various diseases, such as gastroenteritis, upper respiratory tract infections, urinary tract infections, diarrhea and wounds [2]. The essential oil and extracts of *S. khuzistanica* are rich in phenolic compounds, such as carvacrol and free phenolic acids, and especially rosmarinic acid. The species is used in the pharmaceutical and food industries due to its pharmacological and biological properties [3].

Micronutrients, found in small amounts in plants, play an important role in plant growth and development, as well as in the yield and quality of products [4]. Zinc (Zn) is one of the most important micronutrients for plants and acts as a functional, structural or regulatory cofactor for cell membrane stability, chlorophyll, cytochrome, protein and auxin synthesis, the activation of enzymes (dehydrogenases, aldolases, isomerases, transphosphorylases, RNA and DNA polymerases), photosynthesis and cell transparency [4,5,6,7]. It also plays an important role in the transport of metabolites between plant organs. Although Zn is required only in small amounts, its deficiency can cause significant disturbances in the physiological, morphological and metabolic processes in the plant, such as thin leaves, leaf discoloration, short internodes, wilting and necrosis of shoot tips [8]. Zn is essential for spore formation and pollen fertility. Its deficiency reduces the secretion of stigma exudate and prevents fertilization. Therefore, reduction in seed production and fruit set in crops and their yield and quality are promoted by Zn deficiency [7,9].

Boron (B) plays an important role in the physiological processes of crop plants, such as cell elongation, membrane integrity, cell maturation, activation of dehydrogenase enzymes, leaf expansion, development of meristematic tissue, sugar translocation and protein synthesis [10]. In addition, B is an essential element for plant reproductive growth, affecting pollen grain germination, pollen tube elongation and seed and fruit set and yield [7,11]. B deficiency is a common micronutrient problem in the agroecosystems, leading to the abnormal development of reproductive organs, lower yield and poorer crop quality [12,13]. B has also been found to alter essential oil content and the composition of plants [14].

The foliar application of nutrients, especially micronutrients, is a common method that meets the needs of higher plants and is more efficient than soil consumption under unfavorable soil conditions in terms of nutrient availability [15]. Foliar fertilization with Zn and B, in addition to increasing leaf numbers, significantly improves the growth, yield and content of plant nutrients and chemical compounds, including pigments, carbohydrates and the oil concentration in iris [16]. The foliar fertilization of trace elements is suitable for field use and has the advantage of low application rates, good efficacy, uniform distribution and very rapid plant responses [7]. In addition, foliar fertilizers can be designed to meet the specific needs of plants for one or more micronutrients, especially trace elements, and carry out tasks such as correcting deficiencies, strengthening weak or damaged plants, accelerating growth and growing more efficiently [16]. In general, micronutrients applied via foliar spray are just as or even more effective than soil application and result in significantly higher growth and yield [4].

Jahani et al. [17] reported that the total phenolic compounds and essential oil content and yield of *Mentha piperita* L. strongly increase by Zn application. Similarly, Akhtar et al. [18] have reported the essential oil of *M. piperita* to be 28.2% higher by foliar application of 3 ppm Zn chloride. Zn application has significantly increased the length of the peduncle, the number of inflorescence and florets and the length of the main inflorescence, as well as the fresh and dry weight of inflorescences and plants in *Salvia farinacea* L. [19]. The yield and oil content of linseed have significantly increased by B foliar application [20]. In another study on *Brassica napus* L., the spray of B increased seed yield by 46.1% compared to the control. The effect of B fertilizer on seed yield is attributed to the increase in the number of seeds per silique and siliques per plant [21]. Simultaneous foliar application of Zn and B has resulted in the highest plant height, plant dry weight, number of plant branches, 1000-seed weight and seed yield of *Lens culinaris* [22]. The application of Zn and B has significantly increased the number of seeds, the 1000-seed weight and the seed yield in *Arachis hypogaea* L. [23]. The essential oil composition of *Litsea cubeba* has also been significantly affected by the foliar application of Zn and B [24].

Nowadays, the herbal drugs of *S. khuzistanica* are collected from wild niches to meet the demands of the pharmaceutical and food industries. Due to the high demand of the pharmaceutical and food industries for *S. khuzistanica* raw materials and the challenges facing wild collections such as the degradation of genetic diversity, the extinction of ecotypes and the unreliable supply of herbaceous materials, there is an urgent need to cultivate the species in agroecosystems [3]. The main problem associated with the cultivation of the species is low seed production. The growth and essential oil composition of plants in agroecosystems may be affected by various complex factors including genotype, geographical and climatic conditions, soil properties, plant density and nutrient levels [3,25,26]. Regarding the influence of Zn and B on plant growth and development, the aim of the present study was to investigate the effects of the foliar application of Zn and B on the growth, yield, seed production, essential oil content and composition of *S. khuzistanica*.

## 2. Materials and Methods

### 2.1. Plant Materials and Field Experiment

Healthy cuttings of *S. khuzistanica* were taken from a mother plantation grown at the Medicinal and Aromatic Plants Research Garden of Lorestan University, Lorestan province, Iran (altitude 1170 m, longitude 48°26’ E, latitude 33°44’ N), in September 2018. The species was identified by the Department of Botany of the Research Institute of Forests and Rangelands (TARI), Tehran, Iran. A voucher specimen (herbarium No. 58416) has been deposited at the Herbarium of TARI. Cuttings were planted in plastic pots and grown in the greenhouses of the faculty of agriculture, Lorestan University.

A factorial experiment based on the Randomized Complete Block Design with three replications was conducted for two consecutive years (2018 and 2019). The results of farm soil analysis and climatic conditions of the field experiment for both cropping years are presented in Table 1 and Supplementary Table S1, respectively. Small plants of *S. khuzistanica* were hand-planted in the field on 4 April 2018. Each plot (2 × 3 m) contained 4 rows with 50 cm row distance and 50 cm plant distance. All the experimental plots received 50-ton cow manure, 120 kg P as triple superphosphate and 70 kg P as potassium sulfate in March 2018. In both years, 90 kg N was applied as urea in two equal splits, the first dose on 4 May and the second dose 2 months later, in July. During the field experiment, the plants were watered once a week, weeds were controlled by hand hoeing and no pesticide was used. Drip irrigations were provided to the plant at 7-day intervals. Foliar application of B at three concentrations (control or distilled water, 0.4% and 0.8% applied as H_3_BO_3_) and Zn at three concentrations (control or distilled water, 0.3% and 0.6% applied as ZnSO_4_) were carried out. The treatments were applied at three different times—one month after transplanting, three months after transplanting and at the floral budding stage.

### 2.2. Sampling and Measuring Traits

At full flowering stage, five plants were randomly selected from each plot, and yield attributes such as plant height, canopy diameter, number of main branches and number of sub-branches were measured. The aerial parts of selected plants, consisting of the leaves and upper portions of shoots and inflorescences, were harvested in each plot in early October in 2018 and 2019, then air-dried in the shade at room temperature. Plant dry weight was also measured for each plot. The oil-bearing parts of the plant (leaves and flowering shoots) were separated from the woody stems, weighed and recorded as the drug yield of *S. khuzistanica* [27]. The remaining plants in each plot were kept in the field until late November for seed set, then harvested to measure the seed yield. Seed emptiness and 1000-seed weight were also calculated.

### 2.3. Concentration of Zn and B in Leaves

Leaf samples were taken from each plot in early October 2018 and 2019. Samples were rinsed in demineralized water and then air-dried at 70 °C for three days in a forced-air oven [28]. Dried leaves were ground to pass a 30-mesh screen and digested in a ternary solution (HNO_3_:H_2_SO_4_:HCLO_4_ = 10:1:4 with volume). The Zn and B content was determined according to an atomic absorption spectrophotometric method [29]. With the optimized spectroscopic conditions, a steady baseline was recorded. Standard and sample solutions of zinc and boron were aspirated. Spectrograms were recorded by measuring the absorbance at 213.9 nm and 249.8 nm, using boron and zinc lamps, respectively. An air-acetylene flame with fuel flow rate of 1600 mL/min was used. Zn and B concentrations were calculated using the following equation:

$$\mathrm{element}\;\left(\frac{\mathrm{mg}}{\mathrm{kg}}\right)=\frac{\mathrm{titre}\;\mathrm{value}\;\mathrm{from}\;\mathrm{machine}\;\times \;\mathrm{volume}\;\mathrm{used}}{\mathrm{molar}\;\mathrm{mass}}$$

### 2.4. Seed Germination

For the measurement of the seed germination percentage, 75 hand-cleaned seeds were used for each treatment and divided into three replications of 25 seeds. First, seeds were disinfected with 5% sodium hypochlorite for 30 s, then washed with distilled water. Seeds were placed in Petri dishes with filter paper moistened by distilled water. The Petri dishes were kept in a germinator set for a temperature of 25 °C and photoperiod of 16 h. Constant monitoring ensured sufficient moisture throughout the experiment period. The number of seeds germinated as indicated by the radicle emergence was determined every two days. The germination experiment was continued until germinated number became constant. Finally, the seed germination percentage was calculated for each treatment [30].

### 2.5. Essential Oil Isolation

Essential oils from dried samples of *S. khuzistanica* were isolated by hydro-distillation for 3 h (20 g samples were mixed into 400 mL distilled water). The isolated oils were dried over anhydrous sodium sulfate and kept in amber glass vials at 4 °C before analysis. The essential oil content (*w/w*) of each sample was determined from three consecutive replicates and expressed as mg per 100 g sample [31].

### 2.6. GC-FID Analysis and Carvacrol Measurement

The GC-FID analysis of the oils was conducted using a Shimadzu (GC-17A, Kyoto, Japan) gas chromatograph coupled with a flame ionization detector (FID). A DB-5 fused-silica capillary column (30 m × 0.25 mm i.d., film thickness 0.25 mm) was used to separate the essential oil compounds. The oven temperature was increased from 60 to 280 °C at a rate of 4 °C min^−1^ and held isothermal at 250 °C for 10 min. Ion source and transfer-line temperature were 250 °C. Ultra-pure helium was used as the carrier gas with a linear flow rate of 1.1 mL min^−1^. Injector and detector (FID) temperatures were 285 °C. The split ratio was 1:60. All samples and standards were injected three times [31]. The identification of carvacrol was performed by co-injection of samples with authentic standards (Merck, Germany). Quantification was done by GC-FID peak area normalization considering an equal response factor [32].

### 2.7. Extraction Process

Extraction of plant extract was done by maceration method [33]. A total of 15 g of the dried samples was powdered and dissolved in 150 mL of aqueous methanol 80%. Extraction was done by soaking the mixture on a shaker at room temperature for 72 h. The mixture was filtered through filter paper (GF/A, 110 mm; Whatman, Maidstone, UK). The obtained extract was concentrated in a vacuum rotary evaporator at 50 °C, then dried in a vacuum oven at 450 °C for 3 h. The dried extract was used to measure total phenol, total flavonoids and the antioxidant activity assay [25].

### 2.8. Total Phenolic Content

To measure the total phenol content, the Folin Ciocalteu reagent was used [34]. First, 10 μL of each sample extract was mixed with 490 μL distilled water and 500 μL Folin-Ciocalteu reagent. After adding 500 μL saturated sodium carbonate (1%), the mixture was incubated for 2 h at room temperature. Finally, the absorbance of the samples was measured by a UV-vis spectrophotometer (UV-1800) at 765 nm. All samples were analyzed in three replications and the total phenol content was calculated by the standard curve of gallic acid at concentrations of 0, 50, 100, 150, 250 and 500 mg·L^−1^. Total phenolic results were expressed as gallic acid equivalents (mg GAE·g^−1^ plant dry weight).

### 2.9. Total Flavonoid Content

Total flavonoid content was measured by aluminum chloride colorimetric method, according to Quetter-Deleu [35] but with a slight modification. Amounts of 1 mL of the standard extract or solution, 2 mL of 2% aluminum chloride hexahydrate methanolic solution and 6 mL of 5% potassium acetate solution were mixed. After 40 min incubation at 37 °C, the samples were read at 415 nm with a UV-vis spectrophotometer (UV-1800). The flavonoid content of the samples was calculated by plotting standard curve of rutin at concentrations of 0, 50, 100, 150, 250 and 500 mg·L^−1^. The results were expressed in mg of rutin equivalents (RE)·g^−1^ plant dry weight. Samples and standards were analyzed in three replications.

### 2.10. Antioxidant Capacity

#### 2.10.1. DPPH Radical Scavenging Assay

Antioxidant properties of methanolic extract of *S. khuzistanica* were evaluated by DPPH reagent (2,2-dipheny-l-picrylhydrazyl) according to Choi et al. [36]. Different concentrations of each extract (range from 0.01–1.0 mg·mL^−1^) were made in methanol, then 2.5 mL of the extract (methanol for the control) and 1.0 mL of a 3.0 × 10^−4^ M DPPH solution in methanol were mixed and left for 30 min in the dark at 25 °C. The absorbance was recorded at 517 nm (As) using a UV-vis spectrophotometer (UV-1800). Subsequently, the absorbance of a blank solution containing 1.0 mL methanol and 2.5 mL plant extract solution was also recorded. The DPPH solution, plus methanol, were used as the control and ascorbic acid as a positive control. Finally, the antioxidant activity of the extract was expressed based on IC50, indicating a concentration of the extract that results in 50% free radical scavenging.

#### 2.10.2. Ferric-Reducing Antioxidant Power (FRAP) Assay

The FRAP method is based on the reduction of Fe^3+^-TPTZ (yellow) to Fe^2+^-TPTZ (blue) at low pH values. First, fresh FRAP reagent was prepared by mixing 300 mm acetate buffer (pH 3.6), 10 mm TPTZ and 20 mm FeCl_3_·6H_2_O in a ratio of 10:1:1 at 37 °C. Subsequently, 190 µL of FRAP solution was added to 10 µl of methanol extract and kept at 37 °C for eight minutes. The absorbance of the solutions was read at a wavelength of 593 nm using the Bio-Rad (Hercules, CA, USA) microtiter plate reader. A blank reading was taken, and aqueous solutions of ferrous sulphate heptahydrate (25–1000 μM) were used to generate a calibration curve. The antioxidant power of the extract was expressed as μM Fe (II). All tests were performed in triplicate [37].

### 2.11. Data Analysis

Analysis of variance of data was performed based on the studied experimental design with SAS statistical software. The Mean comparison of the treatments were done with the least significant difference experiment at the level of 0.05%.

## 3. Results

### 3.1. Biomass and Yield Attributes

Boron (B) treatment had a significant effect on the fresh and dry weight of plants, the dry stem weight and the drug yield in both years. There was a significant difference in plant height, canopy diameter and the number of main and secondary branches between B treatments in the second year (Supplementary Tables S2 and S3). Foliar application of B significantly increased the fresh and dry weight of plants, the dry stem weight and the drug yield in both years, and the plant height, canopy diameter and the number of main and secondary branches in the second year. The highest fresh weight and dry weight of plants, dry stem weight and drug yield (40.4952 and 85.62 g·plant^−1^ in the first year and second year, respectively) of *S. khuzistanica* were obtained by applying 0.8% B in both years. Application of 0.8% B resulted in 33.32% and 31.94% increase in drug yield compared to control in the first and second year, respectively. Moreover, the highest plant height, canopy diameter, number of main branches and number of secondary branches were obtained by the highest B application in the second year of the experiment (Table 2 and Table 3).

The results of the analysis of variance (Supplementary Tables S2 and S3) showed that the effect of the Zn foliar application on the fresh and dry weight of plants and drug yield was significant in both years. The plant height and number of sub-branches were significantly affected by Zn application in the first year. Moreover, there was a significant difference between the various Zn treatments in the number of main branches and the dry weight of stems in the second year (Supplementary Tables S2 and S3). The highest values for the fresh and dry weight of plants and the drug yield were observed in both years with 0.6% Zn foliar spray. Zn foliar spraying increased the drug yield from the untreated values of 34.095 and 64.05 g·plant^−1^ to 37.808 and 92.61 g·plant^−1^ (10.89% and 44.59%) with the highest dose (0.6% Zn) in the first year and second year, respectively. Foliar fertilization with 0.6% Zn resulted in the highest plant heights, dry stem weights and the number of main branches in the second year (Table 2 and Table 3).

**Figure 1:**
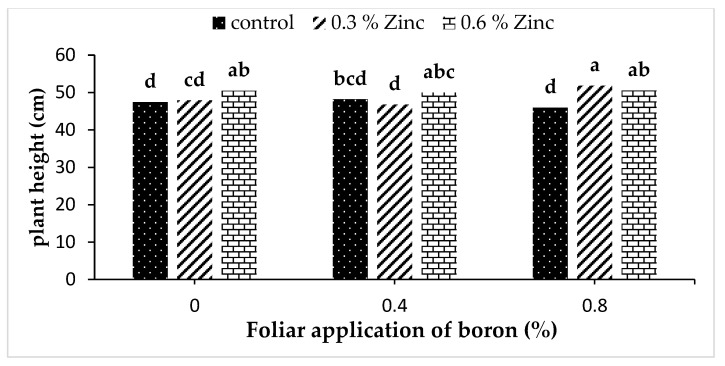
Interaction effect of zinc and boron foliar application on plant height of *S. khuzistanica* in the first year. Means with the same lower-case letter are not significantly different at *p* < 0.05 according to the LSD test.

The interaction of Zn ∗ B was particularly significant for plant height in the first year of the experiment (Supplementary Table S2). The highest plant height (51.86 cm) was obtained in a simultaneous foliar application of 0.3% Zn and 0.8% B (Figure 1).

### 3.2. Seed Traits

The results from the analysis of variance showed significant effects of the B application on seed yield, 1000-seed weight, seed emptiness and seed germination in both years (Supplementary Tables S4 and S5). Seed yield, seed germination and 1000-seed weight were significantly increased by the foliar application of B in both years. The highest values of seed yield, seed germination and 1000-seed weight were found at 0.8% B. Application of 0.8% B resulted in a significant decrease in seed emptiness by 14.16% and 22.37%, as compared to control in the first year and second year, respectively (Table 4 and Table 5).

The seed yield, 1000-seed weight and seed germination were significantly affected by the foliar application of Zn in both years (Supplementary Tables S4 and S5). Application of 0.6% Zn significantly promoted the seed yield (20.85% and 14.96%), 1000-seed weight (11.59% and 10.69%) and seed germination (13.29% and 11.79%), compared to the control in the first year and second year, respectively (Table 4 and Table 5).

### 3.3. B and Zn Concentrations

The results from the analysis of variance showed that the foliar application of B had a positive effect on B content in the leaves of *S. khuzistanica* in both years (Supplementary Tables S4 and S5). The highest B concentration (19.19 and 19.96 mg·kg^−1^ in the first year and second year, respectively) was obtained by 0.8% B spray (Table 4 and Table 5). A significant difference was also observed between Zn treatments in terms of the Zn content of leaves (Supplementary Tables S4 and S5). The application of 0.6% Zn recorded the highest Zn content in the leaf (25.22 and 27.55 mg·kg^−1^ in the first and second years, respectively), while the lowest content was observed in the control in both experimental years (Table 4 and Table 5). On the other hand, the interaction effect of the Zn and B application was not significant for the Zn and B leaf content in any of the experimental years (Supplementary Tables S4 and S5).

### 3.4. Essential Oil Content and Yield and Carvacrol Content

The results from the analysis of variance showed that the foliar application of B had a significant effect on the essential oil content and yield of *S. khuzistanica* in both years (Supplementary Tables S6 and S7). The foliar spray of 0.8% B increased the essential oil content in the leaves of *S. khuzistanica* by 18.6% and 21.58%, compared to the untreated plants in the first year and second year, respectively. Essential oil yield was more affected by the B application than essential oil content. It was 58.05% and 58.33% higher in the 0.8% B treatment than in the first year and second year control, respectively. The 0.8% B treatment resulted in a higher concentration of carvacrol in the essential oil in the first year, while the percentage of carvacrol as the main component of essential oil was significantly higher in 0.8% B plants than in untreated plants. In contrast, in the second year, no significant differences were observed among B treatments in terms of carvacrol content in essential oil (Table 6 and Table 7).

Essential oil content and essential oil yield were significantly affected by zinc application in both years. However, the effect of Zn application on carvacrol content was not significant in any of the experimental years (Supplementary Tables S6 and S7). The highest essential oil content (2.657% and 2.8461%) and yield (5.051 g·m^−2^ and 13.318 g·m^−2^) were obtained with the 0.6% Zn foliar application (Table 6 and Table 7).

### 3.5. Total Phenol and Flavonoid Contents

The results from the analysis of variance showed that the total phenolic content of the extract of *S. khuzistanica* was significantly affected by B application in both years. Significant differences were observed in the total flavonoid content between the different B contents in the second year (Supplementary Tables S6 and S7). The total phenolic content was increased by 34.25% in the first year and 27.5% in the second year by the foliar application of 0.8% B compared to the control. Similarly, total flavonoid content was increased by 22.95% by the application of 0.8% B compared to the control in the second year (Table 6 and Table 7).

The total phenolic content was significantly affected by the Zn application. No significant differences were observed between Zn treatments in the total flavonoid content and the flavonoid to phenolic ratio in both years and the total phenolic content in the second year (Supplementary Tables S6 and S7). The highest content of total phenolics (58.12 mg GAE·g^−1^ dry weight) was obtained under the 0.6% Zn spray in the first year (Table 6).

### 3.6. Antioxidant Capacity

The results indicated that the B application had a significant effect on the antioxidant activity of the *S. khuzistanica* extract calculated by DPPH and FRAP assays in both years (Supplementary Tables S6 and S7). The IC50 (50% reduction in DPPH concentration) value was declined by the foliar application of B in both years, indicating the highest free radical-scavenging activity of the extract in plants treated by 0.8% B. Similarly, the highest FRAP value (742.81 and 831.7 μmol Fe^+2^·g^−1^ dry weight) was observed for application of 0.8% B in the first year and second year, respectively (Table 6 and Table 7).

Various Zn treatments showed significant differences in term of antioxidant capacity in both assays and both years (Supplementary Tables S6 and S7). Application of Zn significantly promoted antioxidant activity of the plant extracts in DPPH assay (decreasing IC50 by the Zn foliar application). The highest (IC50 = 0.0722 and 0.0688 mg·mL^−1^ in the first year and second year, respectively) and the lowest (IC50 = 0.0888 and 0.0788 mg·mL^−1^ in the first year and second year, respectively) antioxidant activity were observed by 0.6% Zn and the control treatment, respectively. Similarly, the highest antioxidant capacity in the FRAP assay (731.25 and 820.14 μmol Fe^+2^·g^−1^ dry weight in the first year and second year, respectively) was obtained by a 0.6% Zn application in both years (Table 6 and Table 7). However, the interaction effect of Zn ∗ B on the antioxidant activity of the extract was not significant in any of the applied assays (Supplementary Tables S6 and S7).

## 4. Discussion

### 4.1. Biomass and Yield Attributes

Zinc plays an important role in the basic processes of cellular mechanism and carbon metabolism, which ultimately affects the growth and development of crops [38]. The results of the present study showed that Zn foliar application significantly increased the growth and yield of *S. khuzistanica* in both years of the experiment. This improvement in the growth and yield of *S. khuzistanica* is possibly due to the involvement of Zn in carbohydrate metabolism, indole acetic acid, RNA and ribosomal function. Our result is similar to the observations made by Wasaya et al. [39] in a study with maize, where they reported that Zn supply increased the growth and yield of crops by increasing the rate of photosynthesis, chlorophyll synthesis and Crop Growth Rate (CGR). Earlier studies demonstrated that Zn foliar application is effective in improving crop yield and quality, while its deficiency reduces crop yield and quality [40,41]. Zn is one of the most important factors affecting the activity of tryptophan synthase. Given the fact that tryptophan acts as a precursor for auxin synthesis, increasing auxin by Zn supply leads to the intensification of apex dominance and consequently increases the longitudinal growth of shoots [42]. In a study on *Petunia hybrida*, it was shown that the application of Zn resulted in increasing plant height, the number of leaves on a plant, the leaf area and leaf chlorophyll content. They concluded that a rise in plant growth and yield could be a result of the Zn-positive role in RNA metabolism and ribosomal content in plant cells, leading to the stimulation of carbohydrates, proteins and DNA synthesis. Zn also contributes to the synthesis of tryptophan as a growth booster [43].

Boron is of the most important essential elements for the higher plants and known to be involved in photosynthesis, N-fixation, meristematic tissue development, respiration, transpiration and other biochemical activities [10,44]. According to our results, the B foliar application increased most of the yield attribute traits of *S. khuzistanica* in both years. A similar result was found for sunflower, where B supply significantly improved plant growth and biomass [45], leading to the conclusion that sunflower growth components, leaf length, crop growth rate (CGR), relative growth rate (RGR), net assimilation rate (NAR) and chlorophyll content were increased by B foliar application. Previous findings have shown that there is a potential interaction between B and plant growth regulators, especially cytokinins. For instance, B deficiency decreased the amount of active cytokinin in *Brassica napus* seedlings [46]. Furthermore, B is one of the structural components of cell wall ingredients, i.e., rhamnogalacturonans, even though its deficiency disrupts cell wall structure and function and reduces the expression of several enzymes located in the cell wall involved in cell expansion, including pectin methylesterase, polygalacturonase and xyloglucan endotransglucosylase. Therefore, it stimulates cells division and elongation, especially in meristematic tissues, and increases plant growth [47].

### 4.2. Seed Traits

Zn acts in flower bud formation and flowering, playing roles in the synthesis of tryptophan as an auxin precursor, and in the translocation of carbohydrates to the site of bud development or to the bud itself [7]. The application of Zn in plants also increases fertility factors such as the formation of spore, fertility of pollen and secretion of stigma exudate, eventually stimulating seed production and fruit set [7,9]. A constant and continuous supply of Zn during plant development could improve the seed yield of the plant possibly due to better plant growth, Zn accumulation in the plant, the photosynthesis rate and enhanced production of phytohormones such as indole acetic acid (IAA), cytokinins and gibberellins [48]. In the present study, foliar application of Zn improved the 1000-seed weight, seed germination and seed yield. A similar effect of Zn application on the grain yield of wheat was also reported by Meena et al [49]. The results of a study by Farooq et al. [50] showed that the seed treatment of bean with Zn improved seed germination, which was attributed to the increase of α-amylase activity. Stimulating effects of the Zn supply on seed yield and germination have also been reported in other plants. For example, examining the impact of different Zn treatments on sunflowers revealed that the highest number of filled seeds and seed yield was obtained in the concentration of 0.5% [45]. Furthermore, it has been reported that the maximum yield and quality of okra seeds were obtained using Zn treatment [44].

According to our results, B had a significant positive effect on seed traits, increased seed yield, 1000-seed weight and seed germination and decreased seed emptiness. Improvement of seed yield and quality was a possible outcome of the improved reproductive growth of plants, carbohydrate metabolism, cell division, germination of pollen grains and the elongation of pollen tubes [7,11]. Azeem et al. [51] reported that the B foliar spray increased seed numbers and the seed weight of cotton through stimulating flowering, facilitating the formation of pollen tubes and increasing seed growth. Likewise, the B application induced the seed germination of *Parthenium argentatum* Gray [52]. B deficiency decreased esterase activity, which is known to be involved in a rupture of stigmatic cuticles for proper pollen tube penetration through stylar tubes for successful fertilization. It has been also observed that the stigmatic exudation that protects stigma from drying, resulting in proper pollen adhesion and germination, is reduced under B deficiency [53]. It could be concluded that the decrease in seed yield under B deficiency is likely to reduce the stigmatic receptivity at the time of anthesis, ultimately causing inhibition in pollen grain viability and resulting in poor germination and seed set/yield [53,54].

### 4.3. Phytochemical Evaluation and Antioxidant Activity

Although Zn application has been shown to affect plant physiology and biochemistry, debates and ambiguities remain about its effects on plant secondary metabolism [55]. In the present study, the effect of Zn application on carvacrol content as the main constituent of the essential oil and total flavonoid was not significant in any years of research. Interestingly, essential oil content and the yield and antioxidant activity of *S. khuzistanica* were significantly increased by the Zn application in both years. On the other hand, the total phenol was significantly risen due to the Zn application in the first year. It seems that Zn could protect the membrane proteins and lipids against free radicals. This may explain the increase in the antioxidant activity of the plant by the foliar application of zinc sulfate [56]. The foliar application of Zn increased phenol, flavonoids and the antioxidant activity of cowpea [57]. The essential oil yield of essential oil-bearing plants depends on the drug yield and essential oil content. Therefore, an increase in essential oil yield due to Zn application was caused by improved drug yield and essential oil content. The increased essential oil content and yield in *S. khuzistanica* as the result of Zn foliar spray are also in line with findings reported by Said-Al Ahl and Mahmoud [58], who found a positive and significant effect of Zn on essential oil content and the yield of *Ocimum basilicum*. Furthermore, Shahhoseini et al. [59] reported the positive effects of Zn on the biomass and terpenoid compounds of *Tanacetum parthenium* essential oil. The foliar application of Zn has improved the quantity and quality of the *S. farinacea* essential oil [19]. In general, Zn is involved in carbon assimilation, saccharide accumulation and scavenging of free radicals, and is overall imposed on photosynthetic carbon metabolism and translocation in relation to secondary metabolite biosynthesis [19,42]. Accordingly, the role of Zn in the carbon allocation to produce phenolic compounds in shikimic acid and acetate pathways can be attributed to this issue [42].

In the current study with *S. khuzistanica*, the total phenol, total flavonoids and antioxidant activity assayed by DPPH and FRAP were increased in response to B. These results were consistent with the findings reported by Thurzo et al. [60] in *Prunus avium* L. and Merino-Gergichevich et al. [61] in *Vaccinium corymbosum* L., who also reported a positive effect of B on the total phenol and flavonoids of studied plants. Given that B has been shown to play an important role in the metabolism of phenolic compounds, the increase in phenol and flavonoids as a result of B application is not surprising [62]. On the other hand, plants with high phenol and flavonoids exhibit high antioxidant activity [27]. In line with these findings, improved antioxidant activities by B application have been reported in other studies by Tavallali et al. [63] and Sarafi et al. [64]. The results of the present study showed the positive impact of B in the content, yield and composition of the *S. khuzistanica* essential oil, which is in accordance with the results reported by Sugier et al. [65] in *Arnica montana* L. and *Arnica chamissonis*. Application of B increased the essential oil content and yield of both *A. montana* and *A.chamissonis*. In addition, their essential oil constituents were altered by the B treatment. Choudhary et al. [66] also reported that the foliar application of B increased the essential oil content of *Mentha arvensis* and *Cymbopogon flexuosus*. They also mentioned that menthol and methyl acetate percentages in the *M. arvensis* essential oil and the citral and geraniol percentages in the *C. flexuosus* essential oil were increased by B application. In general, B fertilization indirectly affects the biosynthesis of primary and secondary metabolites in plants— though different results have been observed in earlier reports. However, in this study, foliar application of B led to an increase in carvacrol, as the predominant component of the *S. khuzistanica* essential oil only in the first year.

## 5. Conclusions

The results of our study suggest that the foliar application of B can be considered a suitable method to increase the quantity and quality of the crop and seed of *S. khuzistanica*. Application of Zn could be a promising method to improve the growth and yield of *S. khuzistanica* as it improves yield attributes, drug yield and phytochemical properties. Zn supply also leads to a higher seed set and better seed germination. The results of the current study indicate that the use of several microelements, such as B and Zn, can improve both the quantity and quality of *S. khuzistanica*. In addition, improving the seed set and seed quality by foliar spraying Zn and B may be useful for growing plants in arid and semi-arid areas. Overall, this study provides some useful information on the effectiveness of the foliar application of Zn and B on *S. khuzistanica* plant production.

## Figures and Tables

**Table 1:** Soil characteristics of the research site.

No	Soil Properties	Unit	No	Soil Properties	Unit
1	Total nitrogen (%)	0.23	9	Organic carbon (%)	1.17
2	Available potassium (mg·kg^−1^)	277	10	Available phosphorus (mg·kg^−1^)	6.6
3	pH	7.7	11	EC (ds/m)	2.03
4	Iron (mg·kg^−1^)	1.71	12	Sand (%)	18.6
5	Magnesium (mg·kg^−1^)	99.4	13	Clay (%)	29.2
6	Zinc (mg·kg^−1^)	0.83	14	Silt (%)	44.2
7	Copper (mg·kg^−1^)	0.41	15	Calcium carbonate (%)	10.8
8	Boron (mg·kg^−1^)	0.41			

**Table 2:** Mean comparison of the simple effects of boron and zinc foliar application on biomass and yield attributes of *S. khuzistanica* in the first year of the experiment.

Treatments		Number of Sub-Branches	Plant Fresh Weight (g·Plant^−1^)	Plant Dry Weight (g·Plant^−1^)	Stem Dry Weight (g·Plant^−1^)	Drug Yield (g·Plant^−1^)	Leaf/Stem
Zinc	Control (0)	35.60 ^b^	139.66 ^b^	58.59 ^b^	24.50 ^a^	34.09 ^b^	1.38 ^a^
	0.3 %	40.86 ^a^	143.41 ^b^	61.63 ^a^	25.36 ^a^	35.49 ^b^	1.40 ^a^
	0.6 %	42.19 ^a^	151.72 ^a^	64.54 ^a^	26.28 ^a^	37.80 ^a^	1.45 ^a^
Boron	Control (0)	40.57 ^a^	125.24 ^c^	54.42 ^c^	24.04 ^b^	30.37 ^c^	1.27 ^b^
	0.4 %	38.27 ^a^	148.66 ^b^	61.82 ^b^	24.74 ^b^	36.52 ^b^	1.48 ^a^
	0.8 %	39.81 ^a^	160.88 ^a^	68.52 ^a^	27.36 ^a^	40.49 ^a^	1.48 ^a^

Means with similar letters in each column, based on an LSD test at a 0.05% probability level, are not significantly different.

**Table 3:** Mean comparison of the simple effects of boron and zinc foliar application on biomass and yield attributes of *S. khuzistanica* in the second year of the experiment.

Treatments		Plant Height (cm)	Canopy Diameter (cm)	Number of Main Branches	Number of Sub-Branches	Plant Fresh Weight (g·Plant^−1^)	Plant Dry Weight (g·Plant^−1^)	Stem Dry Weight (g·Plant^−1^)	Drug Yield (g·Plant^−1^)
Zinc	Control (0)	51.44 ^b^	57.91 ^a^	13.38 ^b^	52.61 ^a^	246.68 ^b^	109.16 ^b^	45.10 ^b^	64.05 ^b^
	0.3 %	53.22 ^ab^	68.61 ^a^	17.11 ^b^	63.56 ^a^	296.54 ^b^	123.89 ^b^	46.76 ^b^	77.12 ^ab^
	0.6 %	55.67 ^a^	69.13 ^a^	22.50 ^a^	62.67 ^a^	363.34 ^a^	157.82 ^a^	65.20 ^a^	92.61 ^a^
Boron	Control (0)	50.11 ^b^	52.05 ^b^	14.33 ^b^	40.50 ^b^	231.29 ^b^	96.43 ^b^	31.53 ^c^	64.89 ^b^
	0.4 %	54.39 ^a^	70.83 ^a^	17.22 ^ab^	63.89 ^a^	306.86 ^a^	138.81 ^a^	55.54 ^b^	83.27 ^a^
	0.8 %	55.83 ^a^	72.77 ^a^	21.44 ^a^	74.44 ^a^	368.41 ^a^	155.62 ^a^	70.00 ^a^	85.62 ^a^

Means with similar letters in each column, based on an LSD test at a 0.05% probability level, are not significantly different.

**Table 4:** Mean comparison of the simple effects of boron and zinc foliar application on seed traits and B and Zn contents of the leaf of *S. khuzistanica* in the first year of the experiment.

Treatments		Seed Yield (g·m^−^^2^)	Seed Germination (%)	1000-Seed Weight (g)	Seed Emptiness (%)	Boron Concentration (mg·kg^−1^)	Zinc Concentration (mg·kg^−1^)
Zinc	Control (0)	0.129 ^c^	60.05 ^b^	1.58 ^b^	22.70 ^a^	13.94 ^a^	15.09 ^c^
	0.3 %	0.144 ^b^	64.21 ^a^	1.73 ^a^	23.11 ^a^	14.35 ^a^	19.85 ^b^
	0.6 %	0.155 ^a^	67.01 ^a^	1.79 ^a^	21.81 ^a^	14.11 ^a^	25.22 ^a^
Boron	Control (0)	0.124 ^c^	57.14 ^c^	1.50 ^c^	23.93 ^a^	9.84 ^c^	19.61 ^a^
	0.4 %	0.138 ^b^	62.87 ^b^	1.74 ^b^	23.16 ^ab^	13.37 ^b^	20.77 ^a^
	0.8 %	0.165 ^a^	71.26 ^a^	1.86 ^a^	20.54 ^b^	19.19 ^a^	19.78 ^a^

Means with similar letters in each column, based on an LSD test at a 0.05% probability level, are not significantly different.

**Table 5:** Mean comparison of the simple effects of boron and zinc foliar application on seed traits and B and Zn contents of the leaf of *S. khuzistanica* in the second year of the experiment.

Treatments		Seed Yield (g·m^−^^2^)	Seed Germination (%)	1000-Seed Weight (g)	Seed Emptiness (%)	Boron Concentration (mg·kg^−1^)	Zinc Concentration (mg·kg^−1^)
Zinc	Control (0)	0.735 ^c^	65.04 ^b^	1.78 ^b^	21.42 ^a^	14.94 ^a^	16.98 ^c^
	0.3 %	0.789 ^b^	70.10 ^a^	1.93 ^a^	21.7 ^a^	15.24 ^a^	22.19 ^b^
	0.6 %	0.845 ^a^	72.01 ^a^	1.99 ^a^	20.08 ^a^	14.89 ^a^	27.55 ^a^
Boron	Control (0)	0.727 ^c^	62.14 ^c^	1.70 ^c^	23.33 ^a^	10.62 ^c^	21.94 ^a^
	0.4 %	0.788 ^b^	68.08 ^b^	1.94 ^b^	21.80 ^a^	14.48 ^b^	23.11 ^a^
	0.8 %	0.854 ^a^	76.92 ^a^	2.06 ^a^	18.11 ^b^	19.96 ^a^	21.67 ^a^

Means with similar letters in each column, based on an LSD test at a 0.05% probability level, are not significantly different.

**Table 6:** Mean comparison of the simple effects of boron and zinc foliar application on phytochemichal traits of *S. khuzistanica* in the first year of the experiment.

Treatments		Essential Oil Content (%)	Essential Oil Yield (g·m^−^^2^)	Carvacrol (%)	Total Phenol (mg GAE·g^−1^ Dry Weight)	FRAP Assay (μmol Fe^+2^·g^−1^ Dry Weight)	DPPH Assay (IC50 mg·mL^−1^)
Zinc	Control (0)	2.05 ^c^	3.52 ^c^	87.38 ^a^	51.77 ^b^	634.98 ^b^	0.088 ^a^
	0.3 %	2.32 ^b^	4.16 ^b^	90.28 ^a^	52.61 ^b^	696.32 ^a^	0.085 ^a^
	0.6 %	2.65 ^a^	5.05 ^a^	88.79 ^a^	58.12 ^a^	731.25 ^a^	0.072 ^b^
Boron	Control (0)	2.15 ^c^	3.29 ^c^	86.52 ^b^	46.03 ^c^	651.14 ^b^	0.095 ^a^
	0.4 %	2.31 ^b^	4.24 ^b^	88.60 ^b^	54.65 ^b^	668.60 ^b^	0.077 ^b^
	0.8 %	2.55 ^a^	5.20 ^a^	91.83 ^a^	61.80 ^a^	742.81 ^a^	0.073 ^b^

Means with similar letters in each column, based on an LSD test at a 0.05% probability level, are not significantly different.

**Table 7:** Mean comparison of the simple effects of boron and zinc foliar application on phytochemical traits of *S. khuzistanica* in the second year of the experiment.

Treatments		Essential Oil Content (%)	Essential Oil Yield (g·m^−^^2^)	Total Phenol (mg GAE·g^−1^ Dry Weight)	Total Flavonoid (mg RE·g^−1^ Dry Weight)	FRAP Assay (μmol Fe^+2^·g^−1^ Dry Weight)	DPPH Assay (IC50 mg·mL^−1^)
Zinc	Control (0)	2.18 ^c^	7.08 ^b^	57.70 ^a^	31.45 ^a^	744.19 ^b^	0.078 ^a^
	0.3 %	2.48 ^b^	9.62 ^b^	60.98 ^a^	32.13 ^a^	772.66 ^b^	0.076 ^ab^
	0.6 %	2.84 ^a^	13.31 ^a^	64.22 ^a^	30.68 ^a^	820.14 ^a^	0.068 ^b^
Boron	Control (0)	2.27 ^c^	7.56 ^b^	53.14 ^b^	27.58 ^b^	736.69 ^b^	0.087 ^a^
	0.4 %	2.47 ^b^	10.49 ^ab^	61.97 ^a^	32.78 ^a^	768.60 ^b^	0.070 ^b^
	0.8 %	2.76 ^a^	11.97 ^a^	67.77 ^a^	33.90 ^a^	831.70 ^a^	0.066 ^b^

Means with similar letters in each column, based on an LSD test at a 0.05% probability level, are not significantly different.

## Data Availability

The datasets used and/or analyzed during the current study are available from the corresponding author on reasonable request.

## Supplementary Materials

The following are available online at https://www.mdpi.com/article/10.3390/plants10112469/s1. Table S1: Climatic conditions of *S. khuzistanica* cultivation area for both cropping years. Table S2: Analysis of variance of the effect of boron and zinc foliar application on biomass and yield attributes of *S. khuzistanica* in the first year of the experiment. Table S3: Analysis of variance of the effect of boron and zinc foliar application on biomass and yield attributes of *S. khuzistanica* in the second year of the experiment. Table S4: Analysis of variance of the effect of boron and zinc foliar application on seed traits and B and Zn contents of leaf of *S. khuzistanica* in the first year of the experiment. Table S5: Analysis of variance of the effect of boron and zinc foliar application on seed traits and B and Zn contents of leaf of *S. khuzistanica* in the second year of the experiment. Table S6: Analysis of variance of the effect of boron and zinc foliar application on phytochemical traits of *S. khuzistanica* in the first year of the experiment. Table S7: Analysis of variance of the effect of boron and zinc foliar application on phytochemical traits of *S. khuzistanica* in the second year of the experiment.
