# Supplementary material for: Improvement of Growth, Yield, Seed Production and Phytochemical Properties of Satureja khuzistanica Jamzad by Foliar Application of Boron and Zinc
Source: Plants (Basel). 2021 Nov 16;10(11):2469. doi: 10.3390/plants10112469 (PMC8620972; doi:10.3390/plants10112469)
Supplement: Supplementary file 1 [file plants-10-02469-s001.zip › plants-1430059-supplementary.pdf]

Table S1. Climatic conditions of *S. khuzistanica* cultivation area for both cropping years.

	**Average Minimum Temperature (°C)**	**Average Maximum Temperature (°C)**	**Average Monthly Temperature (°C)**	**Average Monthly Rainfall (mm)**	**Average Percentage of Relative Humidity**	**Average Minimum Temperature (°C)**	**Average Maximum Temperature (°C)**	**Average Monthly Temperature (°C)**	**Average Monthly Rainfall (mm)**	**Average Percentage of Relative Humidity**
First year	First year									
	April					May				
	7.99	21.83	14.91	6.50	63	11.98	26.38	19.18	1.88	60
	First year									
	June					July				
	16.42	36.64	26.53	0.28	31	21.8	41.62	31.7	0	15
	First year									
	August					September				
	19.66	41.25	30.46	0	15	16.46	36.49	26.47	0.086	19
Second year	Second year									
	April					May				
	6.98	20.28	13.63	6.411	61	12.04	30.67	21.35	0.189	45
	Second year									
	June					July				
	18.16	38.28	28.22	0	24	20.13	40.36	30.24	0	22
	Second year									
	August					September				
	21.29	41.22	31.25	0	19	15.36	36.21	25.78	0	20

Table S2. Analysis of variance of the effect of boron and zinc foliar application on biomass and yield attributes of *S. khuzistanica* in the first year of the experiment.

SOV	Df	Plant Height	Canopy Diameter	Number of Main Branches	Number of Sub-Branches	Plant Fresh Weight	Plant Dry Weight	Stem Dry Weight	Drug Yield	Leaf/ Stem
Replication	2	5.65^{ns}	61.87^{ns}	7.75*	15.47^{ns}	15.26^{ns}	0.19^{ns}	10.07^{ns}	2.79^{ns}	0.55^{ns}
Boron	2	3.05^{ns}	4.33^{ns}	1.02^{ns}	12.29^{ns}	2952.88**	447.59**	27.47*	233.99**	0.137*
Zn	2	22.01**	2.527^{ns}	1.35^{ns}	109.37*	343.02*	79.50**	7.18^{ns}	31.63*	0.13^{ns}
Boron*Zinc	6	11.22*	43.76^{ns}	2.26^{ns}	4.78^{ns}	38.25^{ns}	22.73^{ns}	12.84^{ns}	2.35^{ns}	0.02^{ns}
Error	22	1.81	15.42	0.86	13.29	53.96	7.51	4.924	4.13	0.021

R: replication, ns: not significant; * and ** Significantly at the probability level of P <0.05 and P <0.01, respectively

Table S3. Analysis of variance of the effect of boron and zinc foliar application on biomass and yield attributes of *S. khuzistanica* in the second year of the experiment.

SOV	Df	Plant Height	Canopy Diameter	Number of Main Branches	Number of Sub-Branches	Plant Fresh Weight	Plant Dry Weight	Stem Dry Weight	Drug Yield	Leaf/ Stem
Replication	2	9.25^{ns}	139.46^{ns}	141.36*	483.28^{ns}	8007.46^{ns}	1478.063^{ns}	308.436^{ns}	761.945^{ns}	0.368^{ns}
Boron	2	79.694*	1178.69**	115.11*	2716.028*	42453.48**	8374.498**	3397.227**	1159.578*	1.489^{ns}
Zn	2	40.444^{ns}	360.88^{ns}	188.86**	323.528^{ns}	30837.86**	5603.786**	1119.7**	1840.029*	0.187^{ns}
Boron*Zinc	6	16.222^{ns}	151.97^{ns}	9.263^{ns}	343.347^{ns}	764.8^{ns}	186.21^{ns}	216.919^{ns}	73.71^{ns}	0.37^{ns}
Error	22	15.593	150.45	25.017	507.507	4269.43	736.29	110.177	450.1	0.1278

R: replication, ns: not significant; * and ** Significantly at the probability level of P <0.05 and P <0.01, respectively

Table S4. Analysis of variance of the effect of boron and zinc foliar application on seed traits and B and Zn contents of leaf of *S. khuzistanica* in the first year of the experiment.

SOV	Df	Seed Yield	Seed Germination	1000-Seed Weight	Seed Emptiness	Boron Concentration	Zinc Concentration
Replication	2	0.00054*	0.802^{ns}	0.058*	0.04^{ns}	13.94^{ns}	2.102^{ns}
Boron	2	0.00395**	453.48**	0.2399**	28.492**	200.33**	3.564^{ns}
Zn	2	0.00164*	110.486**	0.0929*	3.945^{ns}	0.37^{ns}	230.98**
Boron*Zinc	6	0.00002^{ns}	28.439^{ns}	0.00715^{ns}	0.876^{ns}	2.62^{ns}	0.688^{ns}
Error	22	0.000086	16.658	0.010328	8.438	6.347	7.713

R: replication, ns: not significant; * and ** Significantly at the probability level of P <0.05 and P <0.01, respectively

Table S5. Analysis of variance of the effect of boron and zinc foliar application on seed traits and B and Zn contents of leaf of *S. khuzistanica* in the second year of the experiment.

Sov	Df	Seed Yield	Seed Germination	1000-Seed Weight	Seed Emptiness	Boron Concentration	Zinc Concentration
Replication	2	2.147 ^{ns}	0.000759 ^{ns}	0.0714**	0.664 ^{ns}	13.85 ^{ns}	2.815 ^{ns}
Boron	2	497.747**	0.03623**	0.294**	64.943**	198.38**	5.248 ^{ns}
Zn	2	116.53**	0.0269**	0.1023**	6.937 ^{ns}	0.3319 ^{ns}	251.41**
Boron*Zinc	6	15.837 ^{ns}	0.00145 ^{ns}	0.0074 ^{ns}	2.739 ^{ns}	2.416 ^{ns}	0.405 ^{ns}
Error	22	14.86	0.000768	0.01026	8.684	5.607	7.4733

R: replication, ns: not significant; * and ** Significantly at the probability level of P <0.05 and P <0.01, respectively

Table S6. Analysis of variance of the effect of boron and zinc foliar application on phytochemical traits of *S. khuzistanica* in the first year of the experiment.

SOV	Df	Essential Oil Content	Essential Oil Yield	Carvacrol	Total Phenol	Total Flavonoid	Flavonoid/ Phenol	FRAP Assay	DPPH Assay
Replication	2	0.0379 ^{ns}	0.268 ^{ns}	26.334 ^{ns}	0.928^{ns}	1.0477^{ns}	0.0855^{ns}	1535.74^{ns}	0.0002**
Boron	2	0.3715**	8.2313**	61.317**	561.098**	63.748^{ns}	0.0169^{ns}	21325.404**	0.0012**
Zn	2	0.8326**	5.2805**	11.344 ^{ns}	107.066*	16.450^{ns}	0.385^{ns}	21376.354**	0.0007**
Boron*Zinc	6	0.01499 ^{ns}	0.0903 ^{ns}	6.12 ^{ns}	36.327^{ns}	24.043^{ns}	0.0706^{ns}	1997.608^{ns}	0.00014^{ns}
Error	2	0.01978	0.07686	9.589	21.653	26.249	0.153	1869.072	0.000052

R: replication, ns: not significant; * and ** Significantly at the probability level of P <0.05 and P <0.01, respectively

Table S7. Analysis of variance of the effect of boron and zinc foliar application on phytochemical traits of *S. khuzistanica* in the second year of the experiment.

SOV	Df	Essential Oil Content	Essential Oil Yield	Carvacrol	Total Phenol	Total Flavonoid	Flavonoid/P henol	FRAP Assay	DPPH Assay
Replication	2	0.0416 ^{ns}	11.627 ^{ns}	2.387 ^{ns}	50.411^{ns}	4.0670ns	0.077^{ns}	1788.521^{ns}	0.00007^{ns}
Boron	2	0.5508**	45.269*	0.242 ^{ns}	488.33**	102.427*	0.013^{ns}	21040.352**	0.00115**
Zn	2	0.9922**	88.462**	0.556 ^{ns}	95.584^{ns}	4.7528^{ns}	0.231^{ns}	13250.352**	0.000248*
Boron*Zinc	6	0.01418 ^{ns}	0.54153 ^{ns}	0.802 ^{ns}	31.795^{ns}	33.976^{ns}	0.0750^{ns}	2824.164^{ns}	0.000098^{ns}
Error	22	0.0251	9.445	0.934	44.520	26.555	0.159	1389.75	0.000070

R: replication, ns: not significant; * and ** Significantly at the probability level of P <0.05 and P <0.01, respectively
